# *Capnocytophaga canimorsus* Septicaemia Presenting With Multiorgan Dysfunction Syndrome (MODS) and Disseminated Intravascular Coagulation (DIC) in an Immunocompetent Individual

**DOI:** 10.1155/crcc/1505682

**Published:** 2026-05-06

**Authors:** Kadison Michel, Prashant Pruthi, Charlie Corke

**Affiliations:** ^1^ Critical Care Unit, Goulburn Valley Health, Shepparton, Australia

**Keywords:** *Capnocytophaga canimorsus*, disseminated intravascular coagulation, multiple organ dysfunction syndrome, purpura fulminans, sepsis

## Abstract

We present a rare case of fulminant *Capnocytophaga canimorsus* septicaemia in a previously well 52‐year‐old immunocompetent woman, following a minor dog‐related forehead abrasion. Despite a recent course of amoxicillin/clavulanate prescribed by her general practitioner, the patient developed profound sepsis characterised by disseminated intravascular coagulation (DIC), multiorgan dysfunction syndrome (MODS) and severe metabolic derangements. Initial symptoms included confusion, diarrhoea and nausea, rapidly progressing to circulatory shock and acute renal failure requiring intubation and continuous renal replacement therapy. Blood cultures later confirmed *C. canimorsus*, and antimicrobial therapy was rationalised accordingly. Radiological evidence revealed bilateral renal cortical necrosis, ultimately resulting in end‐stage renal disease and ongoing dialysis dependency. This case is notable as one of the few documented instances of renal cortical necrosis due to *Capnocytophaga* infection and the only known case in which the patient survived. It highlights the diagnostic and therapeutic challenges of managing rare zoonotic infections, especially in patients without traditional risk factors. Clinicians should maintain a high index of suspicion for *Capnocytophaga* spp. in cases of sepsis of unclear origin, particularly with any history of animal exposure, and involve infectious disease specialists early during care.

## 1. Introduction

*Capnocytophaga canimorsus* is a fastidious Gram‐negative bacillus first described in 1976 by Bobo and Newton [1]. It is a commensal organism in the oral flora of dogs and cats, and human infection typically occurs via animal bites or scratches. Most reported cases of *C. canimorsus* infection occur in individuals with predisposing immunosuppressive conditions such as asplenia, alcoholism, diabetes mellitus, liver cirrhosis or long‐term corticosteroid use [2, 3]. However, this pathogen can also cause fulminant sepsis in immunocompetent hosts. The mortality of *Capnocytophaga* sepsis in previously healthy individuals is estimated to be as high as 30% [2]. We present a case of *C. canimorsus* septicaemia in an immunocompetent woman, notable for its severe presentation with multiorgan dysfunction syndrome (MODS) and disseminated intravascular coagulation (DIC) (Table 1), and a rare complication of bilateral renal cortical necrosis.

**Table 1:** Laboratory investigations on admission demonstrating severe multiorgan dysfunction and disseminated intravascular coagulation.

Investigation	Result	Normal range	Unit
Arterial blood gas			
pH	7.25	7.35–7.45	
pCO_2_	27	35–45	mmHg
PO_2_	62	75–105	mmHg
HCO_3_	12	22–28	mmol/L
Lactate	8.1	0.4–2.0	mmol/L
Electrolytes and metabolites			
Sodium	124	135–145	mmol/L
Potassium	3.0	3.5–5.5	mmol/L
Calcium (ionised)	1.04	1.15–1.30	mmol/L
Magnesium	0.44	0.70–1.10	mmol/L
Phosphate	0.96	0.75–1.50	mmol/L
Renal function			
Urea	12	3.3–7.6	mmol/L
Creatinine	171	45–90 (female)	*μ*mol/L
eGFR	29	> 90	mL/min/1.73m^2^
Liver function tests			
Alanine transaminase (ALT)	149	< 31	U/L
Alkaline phosphatase (ALP)	147	30–110	U/L
Total bilirubin	12	< 17	*μ*mol/L
Albumin	26	35–50	g/L
Haematology			
Haemoglobin	106	115–155	g/L
Platelet count	9	150–400	×10^9^/L
White cell count (WCC)	1.6	4.0–12.0	×10^9^/L
Neutrophil count	1.3	2.0–8.0	×10^9^/L
Coagulation studies			
Prothrombin time (PT)	20.1	11.0–15.0	s
INR	1.9	0.9–1.2	
APTT	64	22–38	s
Fibrinogen	2.1	2.0–4.0	g/L
D‐dimer	54,097	< 500	ng/mL (FEU)
ADAMTS‐13	55.4	40.0–130.0	%
Mixing studies	Corrected	Should correct	
Immunology			
ANA	Positive	Negative	
ANCA	Negative	Negative	
dsDNA	< 10.0	< 10.0	IU/mL
Complement C3	1.04	0.90–1.80	g/L
Complement C4	0.16	0.10–0.40	g/L
Other studies			
Blood film	Schistocytes, inclusions	Normal	
Blood culture	*C. canimorsus*	Negative	
Urine MCS	Negative	Negative	
Imaging	Bilateral renal cortical necrosis	Normal	

*Note:* Comprehensive laboratory values at presentation, highlighting marked metabolic derangements, acute renal and hepatic impairment, profound haematological abnormalities including severe thrombocytopenia and neutropenia and coagulation disturbances consistent with disseminated intravascular coagulation (DIC), reflective of severe *Capnocytophaga canimorsus* septicaemia.

## 2. Case Presentation

A 52‐year‐old woman presented to the emergency department with a 3‐day history of profuse diarrhoea, nausea and confusion. Her partner had called an ambulance when she became acutely disoriented and markedly pale overnight. Her medical history included hypertension, hyperlipidaemia, migraine, depression and previous ischaemic strokes 4 years prior to admission. Home medications included aspirin 100 mg, clopidogrel 75 mg, atorvastatin 80 mg, candesartan 16 mg, venlafaxine XR 150 mg, fremanezumab, sumatriptan nasal spray as needed and topical oestradiol.

On examination, she appeared mottled, pale and cyanotic, with cold extremities. Heart sounds were normal, and breath sounds were clear bilaterally. Her abdomen was soft and nontender. Neurologically, she was confused. Oliguria was noted, progressing to anuria shortly after admission. Notably, there was an abrasion extending to the dermis on her forehead from a dog 1 week prior; her general practitioner had prescribed a 5‐day course of oral amoxicillin–clavulanate after this injury, with 3 days of 875/125 mg twice daily for a weight of 104 kg completed prior to admission.

### 2.1. Investigations

**Figure 1:**
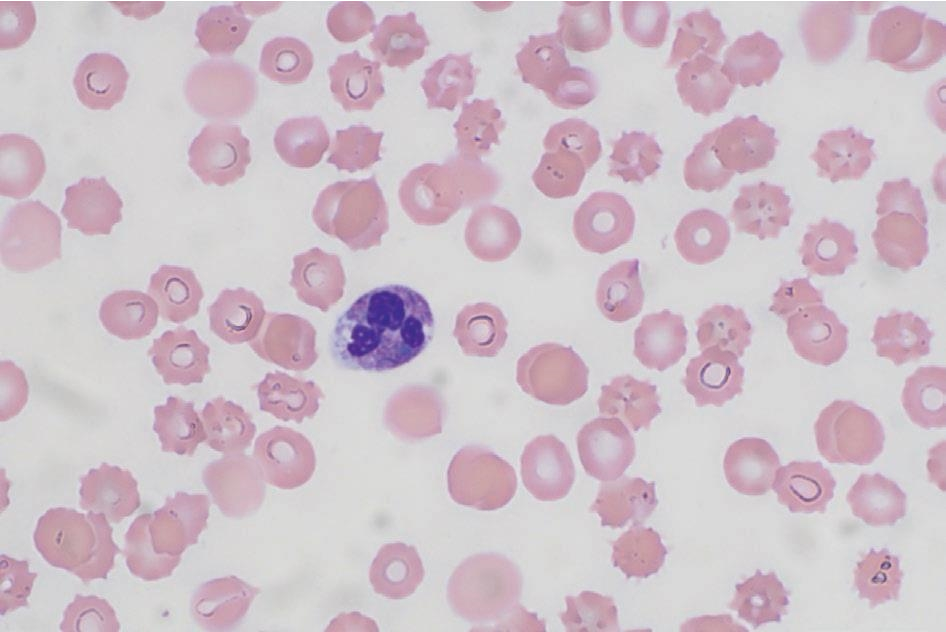
Peripheral blood smear demonstrating bacterial inclusion bodies. Peripheral blood smear at high magnification showing numerous acanthocytes, occasional schistocytes, a rare nucleated red blood cell and marked neutrophilia with left shift and toxic changes. Of particular note, bacterial inclusions are visible within neutrophils, characteristic of severe bacterial sepsis (*Capnocytophaga canimorsus*).

Initial laboratory investigations revealed severe metabolic acidosis (arterial pH 7.25, bicarbonate 12 and lactate 8.1 mmol/L) with an elevated anion gap. There was an acute kidney injury (creatinine 171 *μ*mol/L, urea 12 mmol/L and estimated glomerular filtration rate 29 mL/min/1.73 m^2^) and evidence of liver injury (alanine aminotransferase 149 U/L, alkaline phosphatase 147 U/L and total bilirubin 12 *μ*mol/L). Inflammatory markers were markedly elevated (C‐reactive protein 278 mg/L). A coagulopathy consistent with DIC was present: prothrombin time 20.1 s, international normalised ratio (INR) 1.9, activated partial thromboplastin time (APTT) 64 s, fibrinogen 2.1 g/L and D‐dimer > 50,000 ng/mL (FEU). She had severe thrombocytopenia (platelet count 9 × 10^9^/L) and leucopenia (white cell count 1.6 × 10^9^/L; neutrophils 1.3 × 10^9^/L), indicating bone marrow suppression. A peripheral blood film showed occasional schistocytes and intraneutrophil bacterial inclusion bodies (Figure 1). Given the profound thrombocytopenia, thrombotic microangiopathy was considered; however, ADAMTS‐13 activity was within the normal range, and coagulation studies corrected with mixing argued against thrombotic thrombocytopenic purpura (TTP). Autoimmune serology revealed a low‐titre positive antinuclear antibody (speckled, 1:80) but was otherwise unremarkable (negative extractable nuclear antigens, double‐stranded DNA and ANCA). Given the patient′s confusion, a lumbar puncture was considered to evaluate for meningitis; however, this was contraindicated by her severe coagulopathy and thrombocytopenia, and was not performed.

**Figure 2:**
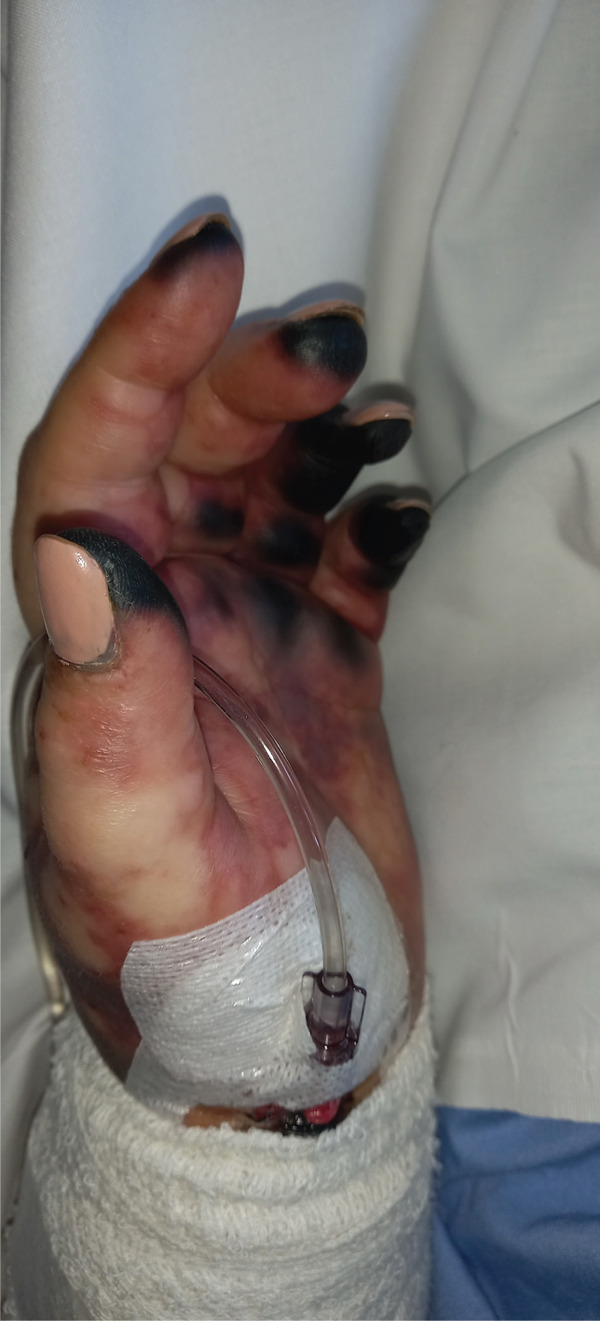
Clinical appearance of purpura fulminans associated with disseminated intravascular coagulation (DIC). Photograph illustrating severe peripheral ischemia and purpuric discolouration of fingers due to purpura fulminans, a manifestation of DIC secondary to fulminant sepsis from *Capnocytophaga canimorsus*. Extensive acral necrosis and cyanosis are evident, underscoring the severe microvascular thrombosis characteristic of this condition.

Microbiological studies and imaging confirmed the diagnosis and extent of organ involvement. Blood cultures taken on admission (before antibiotic administration) grew Gram‐negative bacilli within 24 h. By hospital Day 8, this organism was identified as a *Capnocytophaga* species, and on Day 11, it was confirmed as *C. canimorsus*, consistent with the patient′s exposure to a dog. Urinalysis and urine culture were negative. A whole‐body CT scan demonstrated bilateral renal cortical necrosis, explaining the patient′s oligoanuria and indicating likely progression to permanent renal failure. Cutaneous examination in the intensive care unit (ICU) showed purpuric lesions on the extremities consistent with purpura fulminans (Figure 2).

### 2.2. Differential Diagnosis

Given the patient′s presentation with septic shock, coagulopathy and rash, the initial differential diagnosis was broad. Overwhelming meningococcemia (*Neisseria meningitidis* infection) was a primary consideration due to the DIC and purpuric rash. Other fulminant infections, such as severe Gram‐negative sepsis of intra‐abdominal origin or toxic shock syndrome, were also contemplated. Rickettsial infection (for example, an Australian spotted fever) was considered in the context of rash and systemic collapse. Noninfectious causes of a similar presentation were evaluated, including acute thrombotic microangiopathies like TTP or catastrophic antiphospholipid syndrome. TTP was of particular concern given the profound thrombocytopenia and schistocytes on blood film; however, the normal ADAMTS‐13 activity and presence of a bloodstream infection made TTP unlikely. The recent dog bite and wound infection history raised suspicion for a zoonotic infection. *C. canimorsus* infection was initially considered, but it was deemed less likely given the patient′s recent amoxicillin–clavulanate therapy, which typically covers *Capnocytophaga* species.

### 2.3. Treatment

The patient was rapidly resuscitated with intravenous fluids and vasopressor support. She was intubated for airway protection and mechanical ventilation in the emergency department due to her altered mental status and severe metabolic acidosis, and she was transferred to the ICU for ongoing critical care. Norepinephrine was initiated for vasodilatory shock on Day 0 of ICU admission, and vasopressin was added to achieve the target blood pressure due to refractory vasoplegia. Both vasopressors were successfully weaned off by ICU Day 1 as her haemodynamics stabilised. Continuous renal replacement therapy (CRRT) was started upon ICU admission in light of her anuric acute renal failure and profound acidosis.

Empiric broad‐spectrum antimicrobials were administered early. She was initially started on intravenous ceftriaxone and metronidazole to cover a possible abdominal source, given her gastrointestinal symptoms. With no improvement and ongoing shock, antimicrobial coverage was escalated: meropenem and vancomycin were added, and azithromycin was given to cover atypical pathogens. Once Gram‐negative rods were confirmed in the blood culture (after ~24 h), vancomycin was discontinued. Upon identification of *C. canimorsus*, therapy was streamlined to intravenous piperacillin–tazobactam, which provides appropriate coverage for this organism. Although *C. canimorsus* is typically susceptible to amoxicillin–clavulanate, intravenous piperacillin–tazobactam was used due to the severity of illness and the need for reliable intravenous bactericidal therapy in septic shock, with streamlining guided by infectious diseases and microbiology results. A full 2‐week course of antibiotics was completed. Supportive measures included blood‐product transfusions for DIC and close monitoring of organ function.

### 2.4. Outcome and Follow‐Up

Despite the severity of her illness, the patient gradually improved with intensive treatment. She was successfully weaned off vasopressors and mechanical ventilation, and her coagulopathy and cytopaenias slowly resolved. However, her renal function did not recover; the bilateral cortical necrosis resulted in permanent end‐stage renal failure. She remained dependent on intermittent haemodialysis at hospital discharge. No neurological deficits beyond her initial confusion (which later cleared) were noted upon recovery. The skin lesions from purpura fulminans healed with supportive wound care, leaving residual scarring. At a follow‐up evaluation of 3 months after discharge, the patient was doing well clinically, except for requiring thrice‐weekly dialysis. She has been referred for assessment as a potential renal transplant candidate.

## 3. Discussion

*C. canimorsus* is part of the normal oral flora of dogs and cats and can be transmitted to humans through bites, scratches or even close contact (e.g., licking). Although most severe infections occur in individuals with impaired immune systems or splenic dysfunction, this case demonstrates that immunocompetent individuals are also at risk of life‐threatening disease. Our patient had none of the classic risk factors yet developed fulminant septicaemia with multiorgan dysfunction. Major neurological insults like stroke can induce a temporary immunoparalysis, but her strokes were 4 years prior and thus unlikely to be a factor. Furthermore, comprehensive investigations revealed no underlying immunodeficiency or hematologic disorder.

Published reviews have found that mortality in immunocompetent patients with *Capnocytophaga* sepsis approaches 30% [2]. Early symptoms are often nonspecific, including fever, chills, myalgias and gastrointestinal upset [2]. The infection can progress rapidly to septic shock, accompanied by DIC and purpura fulminans. Infections have also been reported in the absence of an apparent bite or scratch, including meningitis and bacteraemia in a dog owner [4]. In addition, other *Capnocytophaga* species, such as *Capnocytophaga canis*, have been implicated in zoonotic infection, including cellulitis and transient bacteraemia after a cat scratch in an immunocompetent patient [5].

This case is remarkable for the development of bilateral renal cortical necrosis, a rare complication of severe sepsis. Upon reviewing the literature, we identified only one other reported case of *C. canimorsus* sepsis leading to renal cortical necrosis [6]. In that report, the patient did not survive and therefore did not require dialysis. To our knowledge, our patient is the first reported survivor of *C. canimorsus* infection who sustained complete renal cortical necrosis, leaving her dialysis dependent.

An unusual aspect of this case is that the patient had nearly completed a course of amoxicillin–clavulanate before presentation. *Capnocytophaga* species are generally highly susceptible to beta‐lactam antibiotics, including penicillins and beta‐lactam/beta‐lactamase inhibitor combinations [7]. In consultation with the infectious diseases service, an active *Capnocytophaga* infection was initially deemed unlikely given her recent appropriate antibiotic therapy, as *C. canimorsus* is typically susceptible to amoxicillin–clavulanate [7]. One possibility is that the antibiotic was initiated too late or was of insufficient duration to fully eradicate the organism, allowing the infection to progress once therapy was completed or stopped. Alternatively, the severity of the disease may have been such that even a partially effective antibiotic could not prevent the cascade of sepsis and organ failure. This scenario highlights the need for clinical vigilance: a history of appropriate antibiotic prophylaxis should not rule out *Capnocytophaga* in a patient presenting with signs of sepsis after exposure to an animal.

Rapid identification of this pathogen is challenging, as *C. canimorsus* is a slow‐growing and fastidious organism. Matrix‐assisted laser desorption/ionisation time‐of‐flight mass spectrometry (MALDI‐TOF MS) can facilitate earlier identification of fastidious Gram‐negative rods, including *Capnocytophaga* spp., once growth is obtained [8]. In our case, the organism was not definitively identified until 8 days after the hospital stay began. Advanced diagnostic techniques, such as broad‐range 16S rRNA gene PCR or metagenomic sequencing (e.g., nanopore sequencing), have shown promise for rapid identification of *C. canimorsus* in sepsis cases [9]. Early identification can improve outcomes by allowing prompt‐targeted therapy, especially in fulminant cases.

In summary, *C. canimorsus* should be considered in any patient presenting with sepsis and DIC after a dog bite or similar exposure, even if initial antibiotic prophylaxis has been administered. This case highlights that even immunocompetent individuals are not immune to severe infections. Early and aggressive management of sepsis, including the use of appropriate broad‐spectrum antibiotics and critical care support, is paramount. Finally, awareness of this rare but deadly infection and its potential complications, such as purpura fulminans and irreversible organ damage, is essential for clinicians. We urge thorough history‐taking regarding animal exposures in cases of sepsis of unclear origin, as well as early involvement of infectious disease specialists in the care of such patients.

## Funding

No funding was received for this manuscript.

## Disclosure

All manuscript content was written entirely by the authors, who take full responsibility for the accuracy and integrity of the work.

## Consent

Written informed consent was obtained from the patient for publication of this case report and accompanying images.

## Conflicts of Interest

The authors declare no conflicts of interest.

## Data Availability

The data that support the findings of this study are available on request from the corresponding author. The data are not publicly available due to privacy or ethical restrictions.
